# Add-on parsaclisib for patients with myelofibrosis and suboptimal response to ruxolitinib: a randomized phase 3 study

**DOI:** 10.1093/oncolo/oyag201

**Published:** 2026-05-21

**Authors:** Jean-Jacques Kiladjian, Uma Borate, Elisabetta Abruzzese, Valerio De Stefano, Tiejun Gong, Massimo Breccia, Francesca Palandri, Fabrizio Pane, Andrea Patriarca, Hakon Reikvam, Lindsay Rein, Abdulraheem Yacoub, Feng Zhou, Michael Stouffs, Albert Assad, Alessandro Maria Vannucchi

**Affiliations:** Saint-Louis Hospital, Paris Cité University, INSERM, Paris 75010, France; Division of Hematology, The Ohio State University Comprehensive Cancer Center, Arthur G. James Cancer Hospital and Richard J. Solove Research Institute, The James Outpatient Care, Columbus, OH 43210, United States; S. Eugenio Hospital, Tor Vergata University, Rome 00144, Italy; Section of Hematology, Department of Radiological and Hematological Sciences, Catholic University, Fondazione Policlinico Gemelli IRCCS, Rome 00168, Italy; Harbin Institute of Hematology and Oncology, Harbin the First Hospital, Harbin, Heilongjiang 150010, China; Department of Translational and Precision Medicine, Azienda Policlinico Umberto I, Sapienza University, Rome 00161, Italy; Department of Medical and Surgical Sciences, IRCCS Azienda Ospedaliero-Universitaria di Bologna, Bologna 40138, Italy; Department of Clinical Medicine and Surgery, University Federico II, Naples 80131, Italy; Division of Hematology, Department of Translational Medicine, Università del Piemonte Orientale, Novara 28100, Italy; Department of Clinical Science, K.G. Jebsen Center for Myeloid Blood Cancer, University of Bergen, Bergen 5009, Norway; Department of Medicine, Haukeland University Hospital, Bergen, 5009, Norway; Department of Medicine, Duke University School of Medicine, Durham, NC 27710, United States; Department of Internal Medicine, University of Kansas Medical Center, Westwood Campus, Westwood, KS 66205, United States; Incyte Corporation, Wilmington, DE 19803, United States; Incyte Corporation, Wilmington, DE 19803, United States; Incyte Corporation, Wilmington, DE 19803, United States; Department of Experimental and Clinical Medicine, University of Florence, Azienda Ospedaliero-Universitaria Careggi, Florence 50134, Italy

**Keywords:** parsaclisib, primary myelofibrosis, spleen, ruxolitinib

## Abstract

**Background:**

Ruxolitinib (JAK1/JAK2 inhibitor) is indicated for adults with intermediate or high-risk myelofibrosis; however, a subset of patients may exhibit a suboptimal response due to persistent PI3K/AKT activation. The phase 3, randomized, double-blind, placebo-controlled LIMBER-304 study (NCT04551053) investigated the efficacy and safety of add-on parsaclisib (highly selective PI3Kδ inhibitor) in patients with myelofibrosis and suboptimal or declining response to stable ruxolitinib monotherapy.

**Patients and Methods:**

Adults with primary or secondary myelofibrosis who received ruxolitinib with palpable spleen and Myelofibrosis Symptom Assessment Form (MFSAF) total symptom score (TSS) ≥10 were eligible. Primary end point was proportion of patients achieving ≥25% spleen volume reduction (SVR; baseline to week 24); key secondary end point was proportion of patients with ≥50% MFSAF-TSS reduction (baseline to week 24).

**Results:**

In total, 90 patients received parsaclisib/ruxolitinib; 87 received placebo/ruxolitinib. At week 24, 16.7% of patients receiving parsaclisib/ruxolitinib achieved ≥25% SVR vs 9.7% for placebo/ruxolitinib; this difference was not statistically significant. By week 24, ≥50% reduction in MFSAF-TSS was observed in 17.1% of patients receiving parsaclisib/ruxolitinib vs 14.1% for placebo/ruxolitinib. Higher rates of infections (including cytomegalovirus) and gastrointestinal disorders were observed with parsaclisib/ruxolitinib. Grade ≥3 treatment-emergent adverse events occurred in 60.0% of patients receiving parsaclisib/ruxolitinib vs 42.5% with placebo/ruxolitinib. The study was terminated early based on efficacy findings.

**Conclusions:**

Study results suggested adding parsaclisib to stable-dose ruxolitinib was unlikely to offer clinically meaningful benefits. Further research is needed on the potential of JAK and PI3K inhibitor-based combination therapy for patients with myelofibrosis.

Implications for Practice Evidence suggests targeting the PI3K/AKT pathway may have clinically relevant effects in patients with myelofibrosis who experience suboptimal response to the JAK1/JAK2 inhibitor, ruxolitinib. LIMBER-304 evaluated efficacy and safety of adding a highly selective PI3Kδ inhibitor, parsaclisib, in patients with intermediate or high-risk myelofibrosis with suboptimal or declining response to ruxolitinib. Addition of parsaclisib to ruxolitinib suggested improvements in spleen volume reduction and myelofibrosis symptoms, with a manageable safety profile; however, the study was terminated early based on efficacy results. Additional studies are warranted to fully elucidate the role of novel combination therapies for patients with myelofibrosis exhibiting suboptimal response.

## Introduction

Myelofibrosis is a chronic myeloproliferative neoplasm characterized by bone marrow fibrosis, multiorgan extramedullary hematopoiesis (predominantly liver and spleen), and blood cell count abnormalities.^1^^,^^2^ Patients with myelofibrosis present with progressive spleen enlargement and constitutional symptoms including fatigue, unexplained weight loss, night sweats, fever, itching, bone pain, and abdominal discomfort.^1^ Expected survival varies from a median of 14.2 years in patients with a score of 0 (low risk) in the Dynamic International Prognostic Scoring System (DIPSS) to 1.5 years in patients with a score >4 (high risk).^3^ Most patients have dysregulations in the Janus kinase (JAK) signaling pathway, which plays a central role in the regulation of hematopoiesis, making JAK inhibition a therapeutic target for treatment of myelofibrosis.^4^^,^^5^

The selective JAK1 and JAK2 inhibitor ruxolitinib has remained the standard of care for patients with intermediate- or high-risk myelofibrosis^6^ since its first-in-class approval by the US Food and Drug Administration (FDA) in 2011.^7^ Ruxolitinib reduced symptom burden, improved quality of life, and prolonged overall survival (OS) compared with placebo and best available therapy in the COMFORT I and II phase 3 studies.^8–10^ However, some patients have suboptimal response to ruxolitinib or relapse after an initial response, underscoring the need for improved treatments in patients with suboptimal or declining response to ruxolitinib monotherapy.^11^^,^^12^

The phosphoinositide 3-kinase (PI3K)/protein kinase B (AKT) signaling pathway plays a key role in cell growth and survival and is dysregulated in many human cancers. Persistent activation of the PI3K/AKT pathway observed in patients continually treated with ruxolitinib suggests it may drive disease progression in patients with suboptimal response to JAK inhibitors.^13–15^ In preclinical studies, a synergistic antitumor effect has been observed with JAK1/2 plus PI3K inhibitors both in vitro and in vivo,^13–16^ suggesting that combined inhibition of JAK and PI3K/AKT signaling pathways may be a potential treatment strategy for patients with suboptimal or declining response to ruxolitinib.

Several PI3K inhibitors were approved by the FDA in B-cell malignancies, though their use is limited by adverse events (AEs) that appear to be a class effect such as infection, diarrhea, liver problems, rash, and inflammation of the lungs.^17^^,^^18^ Parsaclisib is a next-generation, highly selective PI3Kδ inhibitor that dampens downstream signaling of the PI3K/AKT pathway. Parsaclisib was designed with a unique molecular structure aimed to limit toxicities associated with earlier-generation PI3K inhibitors.^15^ Based on promising preclinical data, a phase 2, open-label study investigated parsaclisib combined with stable doses of ruxolitinib in patients with myelofibrosis and suboptimal response to ruxolitinib. Parsaclisib was associated with reductions from baseline in spleen volume and improvements in symptoms, with manageable toxicity.^19^

The efficacy and safety of parsaclisib added to ruxolitinib in patients with myelofibrosis was further investigated in 2 randomized, double-blind, placebo-controlled phase 3 studies. Here, we report findings from the LIMBER-304 study (NCT04551053),^20^ which assessed efficacy and safety of add-on parsaclisib in patients with myelofibrosis experiencing suboptimal or declining response to ruxolitinib. Results from the LIMBER-313 study (NCT04551066)^21^ evaluating the combination of parsaclisib and ruxolitinib in patients with no prior treatment with JAK or PI3K inhibitors will be reported in a separate publication.

## Methods

### Study design and patients

The global, multicenter, randomized, double-blind, placebo-controlled phase 3 LIMBER-304 study enrolled across sites in Austria, China, Finland, France, Germany, Hungary, Italy, Japan, South Korea, Norway, Poland, Spain, Turkey, the United Kingdom, and the United States. Eligible patients with myelofibrosis were randomized (1:1) to either oral parsaclisib (5 mg once a day) or matching placebo, both added to existing treatment with stable doses of ruxolitinib (5-25 mg twice a day) (Figure S1, see online supplementary material for a color version of this figure). Patients, investigators, and the sponsor remained blinded to treatment throughout the study and block randomization with stratification for baseline platelet count (≥100 × 10^9^/L vs 50 to <100 × 10^9^/L) and DIPSS risk category (high vs intermediate-2 vs intermediate-1) was used. Patients were centrally assigned to treatment using an interactive web/voice response system according to a randomization schedule generated by a sponsor-independent statistician. After 24 weeks, treatment was unblinded and patients could enter an extension period with those receiving placebo crossing over to receive parsaclisib, with continued ruxolitinib treatment, if their hematological parameters were deemed acceptable (platelet count >50 × 10^9^/L and absolute neutrophil count ≥0.5 × 10^9^/L). Treatment could continue for as long as tolerated, until any of the prespecified discontinuation criteria were met.

Eligible patients were men or women aged ≥18 years (≥19 years in South Korea) with a diagnosis of primary or secondary (post–polycythemia vera or post–essential thrombocythemia) myelofibrosis of at least intermediate-1 risk according to DIPSS. Patients had to have been treated with ruxolitinib for ≥3 months with a stable dose (5-25 mg twice a day) for ≥8 weeks before receiving the first dose of study treatment, with evidence of suboptimal or declining response (defined as palpable spleen ≥5 cm below the left subcostal margin and Myelofibrosis Symptom Assessment Form [MFSAF] v4.0 total symptom score [TSS] ≥10 [7-day recall period]) and an Eastern Cooperative Oncology Group Performance Status score of ≤2. Patients were excluded if they had previously been treated with PI3K inhibitors at any time or with an experimental or standard treatment for myelofibrosis (excluding ruxolitinib) in the 3 months prior to starting study treatment, a platelet count <50 × 10^9^/L, recent history of inadequate bone marrow reserve (platelet count <50 × 10^9^/L, absolute neutrophil count <0.5 × 10^9^/L, or peripheral blast count ≥10%), or inadequate liver or renal function at screening.

The study (ClinicalTrials.gov identifier: NCT04551053) was conducted according to the principles of the International Conference on Harmonisation guidelines for Good Clinical Practice, the Declaration of Helsinki and all applicable local laws. Institutional review boards approved the protocol, and all patients provided written informed consent before the start of the study.

### Study end points

The primary end point was the proportion of patients achieving ≥25% reduction in spleen volume from baseline to week 24. The key secondary end point was the proportion of patients with a ≥ 50% reduction from baseline in TSS (measured via MFSAF v4.0 diary) to week 24. Other secondary end points included change from baseline in TSS, time to first ≥50% reduction in TSS (both measured via MFSAF v4.0 diary), OS (determined from the date of randomization until death from any cause), time to the first ≥25% reduction in spleen volume and duration of maintenance of this reduction, and safety and tolerability. Exploratory end points included change in spleen volume and change in spleen size from baseline to each visit where these variables were assessed and number of participants with responses according to the 2013 International Working Group consensus criteria for treatment response in primary and secondary myelofibrosis.^22^

### Assessments

Spleen volume was measured by an independent central reader from magnetic resonance imaging or computed tomography (CT) images obtained during the baseline period (within 7 days before initiation of study treatment), then every 12 weeks until week 108. Spleen length, from the costal margin to the point of greatest splenic protrusion, was assessed by manual palpation at each study visit (baseline, weeks 4, 8, 12, 16, 20, and 24, and every 12 weeks thereafter up to and including the end of treatment visit).

Symptoms of myelofibrosis were assessed using the MFSAF v4.0 diary (24-hour recall period) and included filling up quickly/early satiety, abdominal discomfort/pain, fatigue, night sweats, itching and bone/muscle pains, and pain under left ribs. The diary was completed each night by patients on a handheld electronic device from the first day of baseline (day −7) until the end of week 24.

Safety and tolerability assessments included monitoring treatment-emergent AEs (TEAEs) recorded at each study visit; physical examinations and vital signs performed at screening, baseline, and every study visit from week 4; and electrocardiograms performed at screening, week 24, and end of study/treatment visit. AEs of special interest (AESIs), based on Standardized Medical Dictionary for Regulatory Activities (MedDRA) Queries, when available, or customized aggregates of MedDRA Preferred Terms, were as follows: colitis; diarrhea (grade ≥2); rash (grade ≥2); pneumonitis; dermatitis exfoliative; intestinal perforation; infection with cytomegalovirus (CMV), herpes simplex, herpes (varicella) zoster virus, or *Pneumocystis jirovecii*; alanine aminotransferase increases ≥5 × upper limit of normal (ULN); and aspartate aminotransferase increases ≥5 × ULN.

In patients crossing over from placebo to parsaclisib treatment, additional spleen length, symptoms, and safety and tolerability assessments were carried out at the crossover visit, and 4 weeks post-crossover.

### Statistical methods

A sample size of 212 patients was estimated to provide 95.0% power to detect a statistically significant between-treatment difference in the proportion of patients achieving a ≥ 25% reduction in spleen volume from baseline to week 24 (primary end point), assuming a response rate of 25% for parsaclisib plus ruxolitinib vs 5% with placebo plus ruxolitinib. This sample size would provide 81.3% power to detect a statistically significant between-treatment difference in the proportion of patients with a ≥ 50% reduction from baseline in MFSAF TSS (key secondary end point), assuming a response rate of 35% (25%) for parsaclisib plus ruxolitinib vs 15% (10%) with placebo plus ruxolitinib in patients with normal (low) platelet count at baseline, and 80% power to detect a hazard ratio for OS (secondary end point) of 0.54 with 84 deaths.

Two prespecified nonbinding interim analyses were planned when the first 30% of randomized patients reached week 12 and week 24 assessments of spleen volume and MFSAF TSS or discontinued treatment. Hwang-Shih-DeCani spending functions^23^ with gamma = 2 and gamma = 1 were used to determine the nonbinding futility boundary for spleen volume and MFSAF TSS, respectively. No beta was spent on the first interim analysis.

The primary end point was considered to have been met if a significant between-treatment difference at the 2-sided alpha of 0.05 was observed at the final analysis. If the primary end point was met, the key secondary efficacy end point of proportion of patients with ≥50% reduction in TSS from baseline to week 24 and the secondary end point of OS were tested (in that order) at the 2-sided alpha level of 0.05. Other secondary and exploratory end points were tested using a 2-sided, 5% significance level but not considered alpha-controlled hypotheses.

Efficacy analyses were performed in the intention-to-treat population (all randomized patients). The proportion of patients achieving a ≥ 25% reduction in spleen volume from baseline to week 24 (primary end point) and the proportion of patients with a ≥ 50% reduction from baseline in TSS (key secondary end point) were tested using a Cochran–Mantel–Haenzel test stratified by platelet count at baseline and DIPSS at randomization as described above. Due to insufficient data in the high-risk category, DIPSS stratification factors for the high and intermediate-2 risk categories were combined. All exploratory end points were summarized using descriptive statistics.

Safety analyses were performed in the safety population (ie, all randomized patients who received ≥1 dose of parsaclisib, placebo, or ruxolitinib) and were summarized using descriptive statistics.

## Results

### Patient disposition and characteristics

A total of 177 patients were randomized to receive parsaclisib/ruxolitinib (*n* = 90) or placebo/ruxolitinib (*n* = 87) between May 26, 2021 and March 1, 2023; all randomized patients received at least 1 dose of treatment. Ruxolitinib exposure during the study is summarized in Table S1. The trial was terminated by the sponsor on March 3, 2023, due to futility following the second interim analysis. Full details of patient disposition are summarized in Figure S2 (see online supplementary material for a color version of this figure). Median age at screening was 65 years in the parsaclisib/ruxolitinib group and 63 years in the placebo/ruxolitinib group; most patients had primary myelofibrosis (65.6% and 62.1%, respectively). Median time since diagnosis was lower in the parsaclisib/ruxolitinib group vs the placebo/ruxolitinib group (37.8 vs 56.0 months); all other patient and disease characteristics at screening were balanced between treatment groups (Table 1).

**Table 1:** Patient baseline characteristics.

Characteristic	Parsaclisib/ruxolitinib (*n* = 90)	Placebo/ruxolitinib (*n* = 87)
**Age, median (range), y**	65 (43-81)	63 (33-79)
**Age ≥65 y, %**	52.2	46.0
**Male, %**	58.9	56.3
**Time since first myelofibrosis diagnosis, median (range), mo**	37.8 (2.8-337.1)	56.0 (3.7-233.9)
**Duration of prior ruxolitinib treatment, median (range), days**	673.5 (113.0-4387.0	704.0 (96.0-3105.0)
**Spleen volume, median (range), cm^3^**	1750.3 (373.5-12 023.9)	1791.9 (281.7-5718.3)
**TSS by MFSAF at screening, median (range)**	19 (10-57)	21 (0-63)
**Palpable spleen length, median (range), cm**	10 (4-40)	10 (0-27)
**Myelofibrosis type, %**		
** PMF**	65.6	62.1
** PPV-MF**	15.6	19.5
** PET-MF**	18.9	18.4
**DIPSS risk category, %**		
** Intermediate-1**	44.4	50.6
** Intermediate-2**	44.4	43.7
** High**	11.1	5.7
**Had prior transfusions of pRBC, %**	27.8	31.0

Abbreviations: DIPSS, Dynamic International Prognostic Scoring System; MFSAF, Myelofibrosis Symptom Assessment Form; mo, months; PET-MF, post–essential thrombocythemia myelofibrosis; PMF, primary myelofibrosis; PPV-MF, post–polycythemia myelofibrosis; pRBC, packed red blood cells; TSS, total symptom score; y, years.

### Spleen volume and palpable spleen length

**Figure 1.**
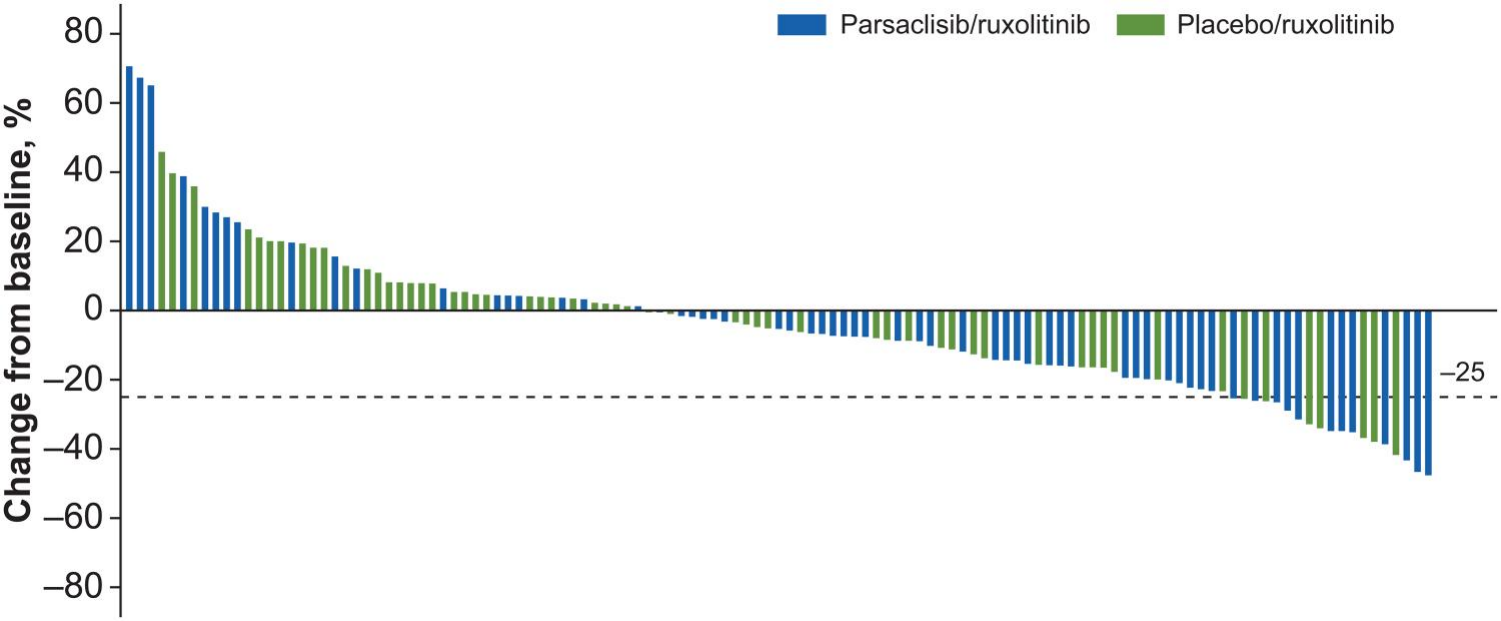
Change in spleen volume in patients with myelofibrosis. Percentage change in spleen volume from baseline to week 24 (individual patient data; *n* = 121). The dotted line represents a 25% decrease in spleen volume from baseline.

**Figure 2.**
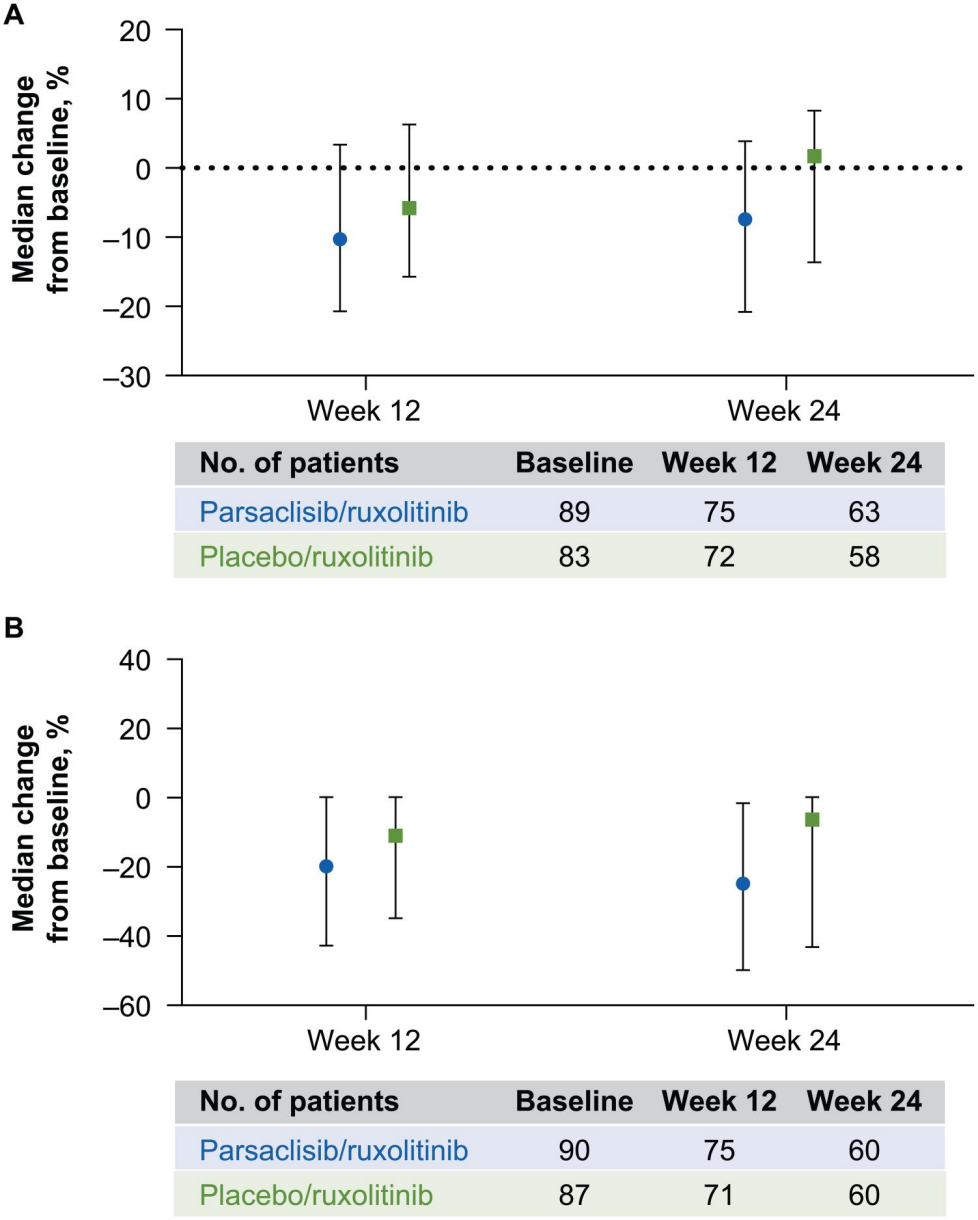
Median change in spleen volume and spleen length in patients with myelofibrosis. Median percentage change from baseline in (A) spleen volume and (B) spleen length at all time points evaluated up to week 24. Error bars represent the interquartile range.

At week 12, the percentage of patients with a reduction in spleen volume ≥25% was numerically greater with parsaclisib/ruxolitinib compared with placebo/ruxolitinib (14.5% [12/83 patients] vs 6.3% [5/80 patients]). However, by week 24 (primary end point), the between-treatment difference had decreased and did not reach statistical significance (16.7% [12/72 patients] vs 9.7% [7/72 patients]; Figure 1). Exploratory end points of change from baseline in spleen volume or spleen length over time showed no significant differences between treatment groups (Figure 2). Time to the first ≥25% reduction in spleen volume and duration of maintenance of this reduction (both secondary end points) could not be estimated as too few participants had a reduction in spleen volume ≥25%.

### Change in myelofibrosis symptoms

**Figure 3.**
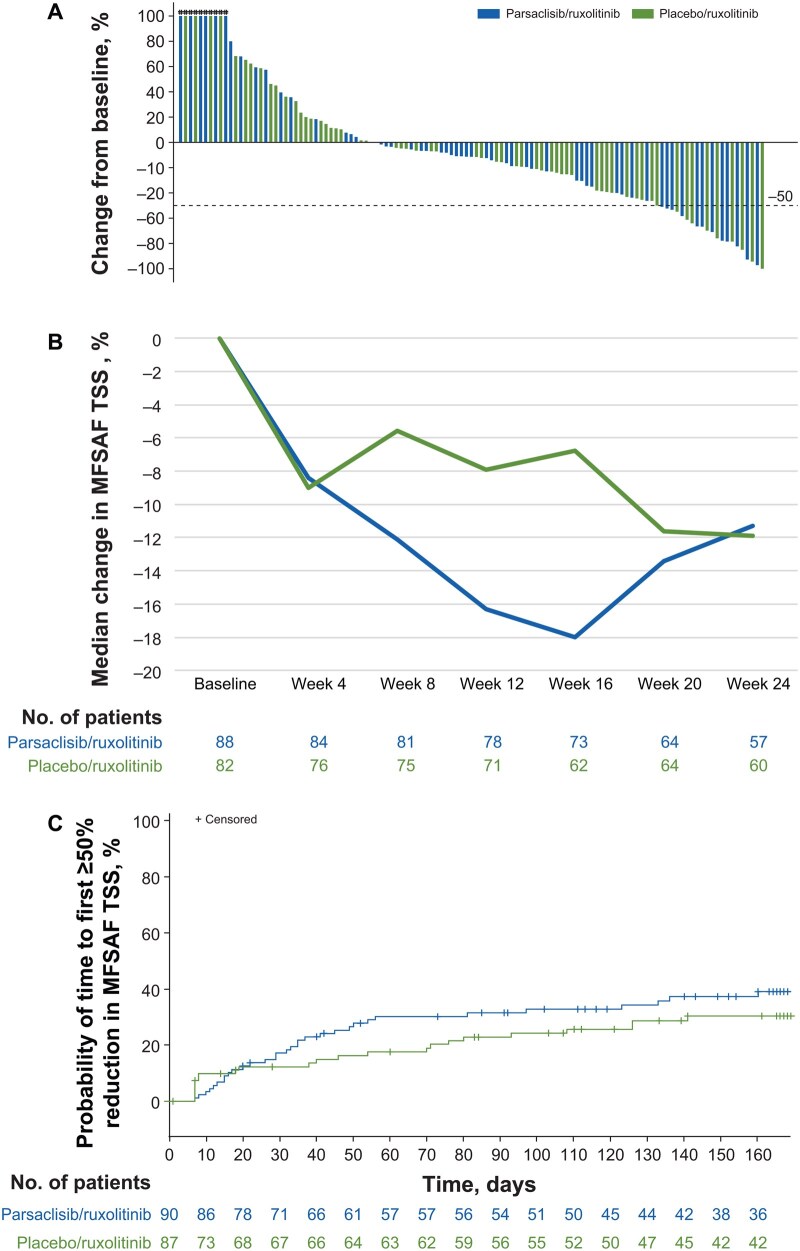
Change in MFSAF TSSs from baseline and likelihood of achieving a ≥50% reduction from baseline in MFSAF TSS in patients with myelofibrosis. (A) Percentage change in MFSAF TSS from baseline to week 24 (individual patient data; *n* = 117). Dotted line represents a 50% decrease in MFSAF TSS from baseline. (B) Median percentage change in MFSAF TSS from baseline. (C) Kaplan-Meier estimates of time to first ≥50% reduction in MFSAF TSS from baseline. Hashtags indicate patients with >100% percent change in MFSAF TSS from baseline. Tick marks indicate censored data. MFSAF, Myelofibrosis Symptom Assessment Form; TSS, total symptom score.

**Figure 4.**
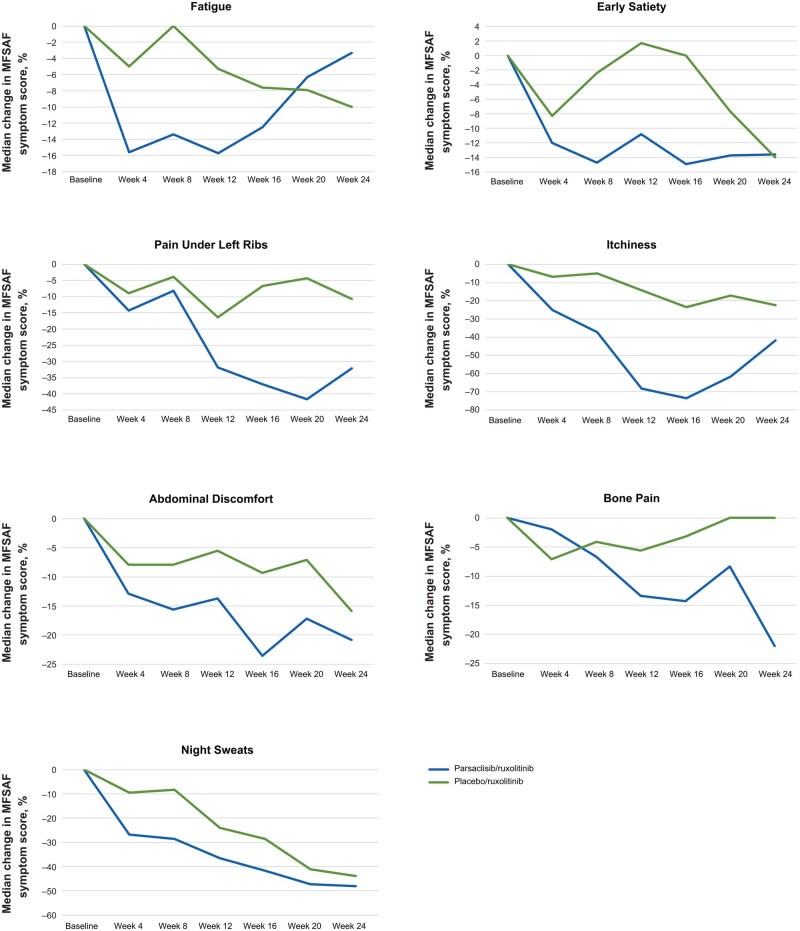
Change in MFSAF score from baseline for individual symptoms. Median percentage change in MFSAF scores from baseline to week 24 for each of the individual symptoms assessed. MFSAF, Myelofibrosis Symptom Assessment Form.

There was a trend with a numerically higher percentage of patients with a reduction in MFSAF TSS ≥50% from baseline with parsaclisib/ruxolitinib vs placebo/ruxolitinib at week 12 (exploratory end point; 17.9% [15/84 patients] vs 8.6% [7/81 patients]). Like effects seen with spleen volume, by week 24 (key secondary end point), the between-treatment difference had decreased and did not reach statistical significance (17.1% [12/70 patients] vs 14.1% [10/71 patients]; Figure 3A). Similarly, median MFSAF TSS appeared to decrease more from baseline to weeks 8, 12, and 16 in the parsaclisib/ruxolitinib group compared with the placebo/ruxolitinib group, although differences were no longer observed by weeks 20 and 24 (key secondary end point; Figure 3B). Patients randomized to parsaclisib/ruxolitinib also demonstrated a higher cumulative probability of achieving a 50% reduction in MFSAF TSS compared with those receiving placebo/ruxolitinib from day 20 to end of treatment; however, these differences were not significant (Figure 3C). Similar trends were observed in individual symptoms, with greater decreases from baseline in the parsaclisib/ruxolitinib group compared with the placebo/ruxolitinib group seen in most symptoms from week 4 (Figure 4).

### Overall survival and treatment response per International Working Group consensus criteria

Due to the early termination of the study, follow-up time was not long enough to estimate median OS. No patients in either group experienced a complete response. Partial response (PR) was experienced by 5 (5.6%) patients in the parsaclisib/ruxolitinib; no patients achieved PR in the placebo/ruxolitinib group. A total of 20 (22.2%) patients in the parsaclisib/ruxolitinib group and 11 (12.6%) in the placebo/ruxolitinib group experienced clinical improvement. Stable disease was observed in 37 (41.4%) and 51 (58.6%) patients, and progressive disease occurred in 3 (3.3%) and 1 (1.1%) patients in the parsaclisib/ruxolitinib and placebo/ruxolitinib groups, respectively; disease progression assessments were missing for 25 (27.8%) and 24 (27.6%) patients, respectively.

### Translational assessments

JAK2 V617F was the most represented driver mutation in both treatment groups. Consistent with the clinical findings for the primary and secondary end points, no statistically significant differential effects were observed for the median reduction in JAK2 V617F variant allele frequency at week 24 vs baseline (−0.80% and 0.04% for the parsaclisib/ruxolitinib and placebo/ruxolitinib groups, respectively).

### Safety and tolerability

Any-grade TEAEs were experienced by 81/90 (90.0%) patients in the parsaclisib/ruxolitinib group, 76/87 (87.4%) in the placebo/ruxolitinib group, and 33/41 (80.5%) in the crossover cohort after week 24 (Table 2). The most common any-grade TEAEs occurring more frequently (>5%) with parsaclisib/ruxolitinib vs placebo/ruxolitinib included anemia (31.1% vs 25.3%), platelet count decreased (24.4% vs 16.1%), COVID-19 (21.1% vs 11.5%), pyrexia (15.6% vs 9.2%), CMV infection (11.1%), thrombocytopenia (11.1% vs 10.3%), diarrhea (11.1% vs 10.3%), and pneumonia (10.0% vs 3.4%) (Table 2). Grade ≥3 TEAEs were reported more often with parsaclisib/ruxolitinib (54/90; 60.0%) than placebo/ruxolitinib (37/87; 42.5%) and the crossover cohort (18/41; 43.9%) (Table 3). Most frequently reported (>5%) grade ≥3 TEAEs with parsaclisib/ruxolitinib were anemia (22.2%), platelet count decreased (16.7%), thrombocytopenia (10.0%), pneumonia (7.8%), and COVID-19, and COVID-19 pneumonia (5.6% each). Most common grade ≥3 TEAEs were generally consistent with those reported with placebo/ruxolitinib, except there was a higher incidence (>5%) of platelet count decreased (16.7% vs 5.7%), pneumonia (7.8% vs 2.3%), and COVID-19 (5.6% vs 0) with parsaclisib/ruxolitinib vs placebo/ruxolitinib (Table 3).

**Table 2:** Most common TEAEs of any grade occurring in ≥5% of patients in any treatment group.

Event, *n* (%)	Parsaclisib/ruxolitinib (*n* = 90)	Placebo/ruxolitinib (*n* = 87)	Crossover from placebo to parsaclisib/ruxolitinib (*n* = 41)
**Hematological**			
** Anemia**	28 (31.1)	22 (25.3)	6 (14.6)
** Platelet count decreased**	22 (24.4)	14 (16.1)	6 (14.6)
** Thrombocytopenia**	10 (11.1)	9 (10.3)	8 (19.5)
** White blood cell count decreased**	8 (8.9)	7 (8.0)	0 (0.0)
** Neutrophil count decreased**	5 (5.6)	3 (3.4)	1 (2.4)
** Neutropenia**	0 (0.0)	1 (1.1)	3 (7.3)
**Nonhematological**			
** COVID-19**	19 (21.1)	10 (11.5)	7 (17.1)
** Pyrexia**	14 (15.6)	8 (9.2)	1 (2.4)
** CMV infection**	10 (11.1)	0 (0.0)	2 (4.9)
** Diarrhea**	10 (11.1)	9 (10.3)	1 (2.4)
** Pneumonia**	9 (10.0)	3 (3.4)	3 (7.3)
** Abdominal pain upper**	8 (8.9)	2 (2.3)	0 (0.0)
** ALT increased**	8 (8.9)	3 (3.4)	4 (9.8)
** Cough**	8 (8.9)	4 (4.6)	2 (4.9)
** Dizziness**	8 (8.9)	2 (2.3)	1 (2.4)
** Headache**	8 (8.9)	4 (4.6)	1 (2.4)
** Nausea**	8 (8.9)	7 (8.0)	2 (4.9)
** AST increased**	7 (7.8)	4 (4.6)	4 (9.8)
** Asthenia**	7 (7.8)	4 (4.6)	3 (7.3)
** Hyperuricemia**	7 (7.8)	4 (4.6)	0 (0.0)
** Constipation**	6 (6.7)	0 (0.0)	0 (0.0)
** Dyspnea**	6 (6.7)	3 (3.4)	0 (0.0)
** Stomatitis**	6 (6.7)	2 (2.3)	1 (2.4)
** COVID-19 pneumonia**	5 (5.6)	1 (1.1)	0 (0.0)
** Epistaxis**	5 (5.6)	3 (3.4)	0 (0.0)
** Pruritus**	5 (5.6)	6 (6.9)	0 (0.0)
** Arthralgia**	4 (4.4)	3 (3.4)	3 (7.3)
** Back pain**	4 (4.4)	5 (5.7)	0 (0.0)
** CMV test positive** ^a^	4 (4.4)	0 (0.0)	3 (7.3)

Abbreviations: ALT, alanine transaminase; AST, aspartate aminotransferase; CMV, cytomegalovirus; TEAE, treatment-emergent adverse event.

a Decision to report positive CMV tests as adverse events was based on investigator judgement, using results from either local or central testing.

**Table 3:** TEAEs of grade ≥3 occurring in at least 2 patients in any treatment group.

Event, *n* (%)	Parsaclisib/ruxolitinib (*n* = 90)	Placebo/ruxolitinib (*n* = 87)	Crossover from placebo to parsaclisib/ruxolitinib (*n* = 41)
**Hematological**			
** Anemia**	20 (22.2)	16 (18.4)	4 (9.8)
** Platelet count decreased**	15 (16.7)	5 (5.7)	4 (9.8)
** Thrombocytopenia**	9 (10.0)	5 (5.7)	6 (14.6)
** Neutrophil count decreased**	3 (3.3)	2 (2.3)	0 (0.0)
** White blood cell count decreased**	3 (3.3)	3 (3.4)	0 (0.0)
** Lymphocyte count decreased**	2 (2.2)	2 (2.3)	0 (0.0)
** Leukocytosis**	0 (0.0)	2 (2.3)	0 (0.0)
** Neutropenia**	0 (0.0)	1 (1.1)	2 (4.9)
**Nonhematological**			
** Pneumonia**	7 (7.8)	2 (2.3)	2 (4.9)
** COVID-19**	5 (5.6)	0 (0.0)	0 (0.0)
** COVID-19 pneumonia**	5 (5.6)	1 (1.1)	0 (0.0)
** Pneumonia (influenza)**	2 (2.2)	0 (0.0)	0 (0.0)

Abbreviation: TEAE, treatment-emergent adverse event.

A higher proportion of patients in the parsaclisib/ruxolitinib group were reported to have at least 1 serious TEAE (33/90; 36.7%) than in the placebo/ruxolitinib group (15/87; 17.2%). Serious AEs experienced by at least 2 patients in any group generally occurred in a higher number of patients receiving parsaclisib/ruxolitinib than those receiving placebo/ruxolitinib: pneumonia (*n* = 8; 8.9% vs *n* = 2; 2.3%), COVID-19 pneumonia (*n* = 5; 5.6% vs *n* = 1; 1.1%), COVID-19 (*n* = 3; 3.3% vs 0), anemia (*n* = 2; 2.2% vs *n* = 2; 2.3%), platelet count decreased (*n* = 2; 2.2% vs 0); pyrexia (2.2% vs 0), and influenza pneumonia (*n* = 2; 2.2% vs 0; Table S2) and were most frequently reported in the MedDRA system organ class of “infections and infestations” (*n* = 20; 22.2% vs *n* = 5; 5.7%). A higher proportion of patients receiving parsaclisib/ruxolitinib experienced at least 1 serious AE considered by the investigator to be related to treatment than the placebo/ruxolitinib group (*n* = 13; 14.4% vs *n* = 6; 6.9%). The most frequently reported (>2%) serious AEs considered related to parsaclisib or placebo treatment were pneumonia (*n* = 5; 5.6%) and platelet count decreased (*n* = 2; 2.2%) in the parsaclisib/ruxolitinib group and anemia (*n* = 2; 2.3%) in the placebo/ruxolitinib group. A similar incidence of all AESIs occurred in both groups (Table S3), except for CMV infection, which occurred in 10 (11.1%) patients receiving parsaclisib/ruxolitinib and 0 patients receiving placebo/ruxolitinib. When evaluated by grouped terms, AESIs occurred more frequently (>5%) in the parsaclisib/ruxolitinib group than in the placebo/ruxolitinib group for diarrhea (16.7% vs 11.5%), rash (11.1% vs 3.4%), pneumonia (38.9% vs 20.7%), and CMV infection (18.9% vs 0%).

Eleven fatal AEs were reported during the study: 6 occurred with parsaclisib/ruxolitinib (COVID-19 pneumonia, *n* = 3; and enterocolitis infectious, pneumonia [with concurrent multiple organ dysfunction syndrome], and influenza pneumonia, *n* = 1 each), 3 with placebo/ruxolitinib (subdural hematoma, breast neoplasm, and splenic hemorrhage, *n* = 1 each), and 2 in the crossover cohort (subdural hematoma and bronchopulmonary aspergillosis, *n* = 1 each). No fatal AE was deemed related to study treatment by the investigator.

In the crossover cohort, 1 life-threatening AE of necrotizing colitis was reported.

## Discussion

LIMBER-304 investigated the efficacy, safety, and tolerability of add-on parsaclisib in patients with myelofibrosis with suboptimal or declining response to stable-dose ruxolitinib monotherapy. Results at week 12 indicated a trend toward improved spleen volume responder rates (ie, percentage of patients with a decrease in spleen volume of ≥25%) with the addition of parsaclisib vs placebo, although this did not reach statistical significance. However, the between-treatment difference in spleen volume responder rates was not maintained at week 24 (primary end point) due to a higher-than-expected response in the placebo/ruxolitinib group (<5% expected response vs 9.7% observed). A similar pattern was observed for changes in symptoms over time, with an initial trend toward improvement at 12 weeks in the parsaclisib/ruxolitinib group that was not maintained at 24 weeks, again, due to higher-than-expected responses in the placebo/ruxolitinib group. The incidence of TEAEs was generally similar between the 2 treatment groups, although the incidence of serious, severe, and fatal TEAEs was higher with parsaclisib/ruxolitinib. None of the fatal AEs that occurred during the study were deemed related to treatment by the investigator. The incidence and severity of infections and gastrointestinal (GI) disorders tended to be higher with parsaclisib/ruxolitinib, consistent with known PI3K inhibitor class effects.^17^^,^^18^ Based on efficacy findings, the study was terminated following the advice of an independent data monitoring committee.

A spleen volume reduction of ≥35% is accepted as the cutoff for response in patients who have not previously received treatment with JAK inhibitors such as ruxolitinib.^9^^,^^10^ However, data from clinical studies of new agents involving patients previously treated with ruxolitinib suggest this cutoff is unrealistic for patients with treatment-resistant disease, reporting a relatively small percentage of patients achieving reductions of this magnitude.^12^^,^^24^^,^^25^ The present study was conducted in a difficult-to-treat population of patients who were experiencing suboptimal or declining response to long-term treatment with ruxolitinib. As such, the smaller cutoff for response (reductions of ≥25%) used in LIMBER-304 was regarded as appropriate, given that spleen volume reductions as low as 10% have been shown to improve prognosis in patients with myelofibrosis.^26^

After 12 and 24 weeks of parsaclisib/ruxolitinib treatment, 14.5% and 16.7% of patients, respectively, achieved spleen volume reductions of ≥25%. These are lower response rates than those from a previous phase 2 study in a similar patient population, where daily parsaclisib added to ruxolitinib was associated with response rates of 22% and 28% at weeks 12 and 24, respectively.^19^ Patients in both the phase 2 and the LIMBER-304 study had been heavily pretreated; this may have been a factor in the modest responses in spleen volume reductions observed in both studies.^19^

In our study, the initial trend to higher spleen volume and MSAF TSS responder rates with parsaclisib vs placebo added to stable-dose ruxolitinib observed at 12 weeks did not continue at 24 weeks. This could be a result of the emergence of adaptative mechanisms following pharmacological PI3K signaling pathway inhibition, a phenomenon that has been reported in studies of solid tumors and can eventually lead to drug resistance.^27^^,^^28^ Better understanding of the role PI3K signaling plays in myelofibrosis could inform additional targets within this pathway that could be inhibited simultaneously to counteract an adaptative response.

In conclusion, findings from the interim analysis of the LIMBER-304 study suggested adding parsaclisib to stable-dose ruxolitinib was unlikely to provide clinically meaningful benefits in heavily pretreated patients with myelofibrosis and suboptimal or declining response to ruxolitinib, resulting in study termination. The overall incidence of TEAEs was similar between the 2 treatment groups, but there was a greater incidence of serious, severe, and fatal TEAEs, as well as infection and GI disorders with the parsaclisib/ruxolitinib combination. Given the overall findings, close monitoring for infections and toxicities and reassessment of benefit-risk for patients on combination therapy is warranted. Further investigations may clarify if PI3K inhibitors could have a role in selected patient subsets, or in alternative combinations, as potential therapeutic strategies in myeloproliferative neoplasms.

## Supplementary Material

oyag201_Supplementary_Data

## Data Availability

Incyte Corporation (Wilmington, DE) is committed to data sharing that advances science and medicine while protecting patient privacy. Qualified external scientific researchers may request anonymized data sets owned by Incyte for the purpose of conducting legitimate scientific research. Researchers may request anonymized data sets from any interventional study (except phase 1 studies) for which the product and indication have been approved on or after January 1, 2020, in at least one major market (eg, United States, European Union, and Japan). Data will be available for request after the primary publication or 2 years after the study has ended. Information on Incyte’s clinical trial data-sharing policy and instructions for submitting clinical trial data requests are available at: https://www.incyte.com/Portals/0/Assets/Compliance%20and%20Transparency/clinical-trial-data-sharing.pdf? ver=2020-05-21-132838-960
