# Supplementary material for: Add-on parsaclisib for patients with myelofibrosis and suboptimal response to ruxolitinib: a randomized phase 3 study
Source: Oncologist. 2026 May 21;31(7):oyag201. doi: 10.1093/oncolo/oyag201 (PMC13257860; doi:10.1093/oncolo/oyag201)
Supplement: oyag201_Supplementary_Data [file oyag201_supplementary_data.pdf]

Supplemental Materials

Add-on parsaclisib for patients with myelofibrosis and suboptimal response to ruxolitinib: a randomized phase 3 study

Jean-Jacques Kiladjian, Uma Borate, Elisabetta Abruzzese, Valerio De Stefano, Tiejun Gong, Massimo Breccia, Francesca Palandri, Fabrizio Pane, Andrea Patriarca, Hakon Reikvam, Lindsay Rein, Abdulraheem Yacoub, Feng Zhou, Michael Stouffs, Albert Assad, Alessandro Maria Vannucchi

Supplemental Figure 1. Study design.

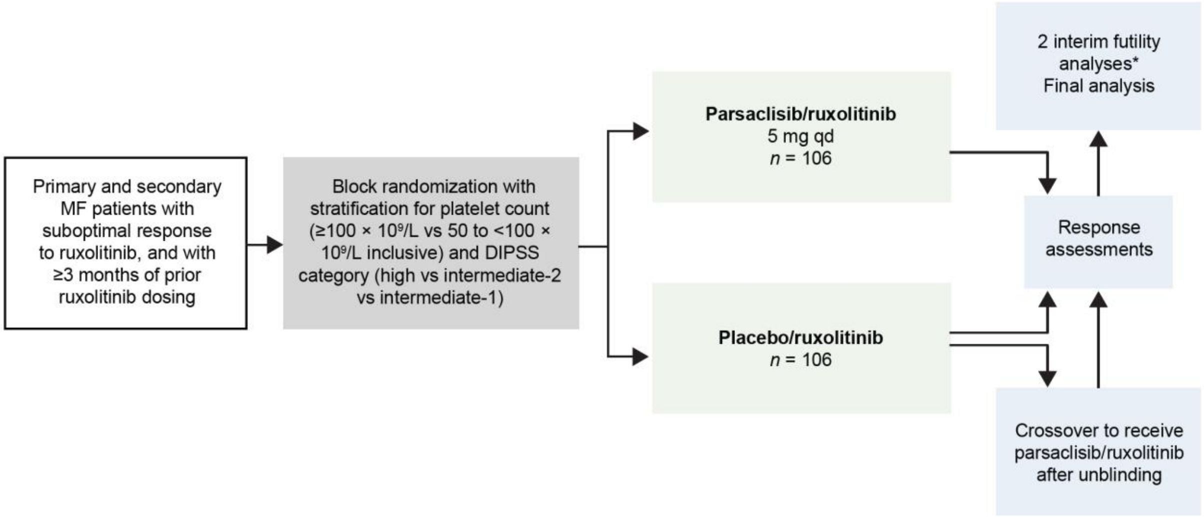

*Based on the results of the first interim analysis, the data monitoring committee could recommend study continuation with no changes to enrollment, continuation with no further enrollment, or termination. After the second interim analysis, a recommendation to continue or terminate the study could be made.

DIPSS, Dynamic International Prognostic Scoring System; JAK, janus kinase; MF, myelofibrosis; PI3K, phosphatidylinositol 3-kinase; QD, once daily.

Supplemental Figure 2. Patient disposition

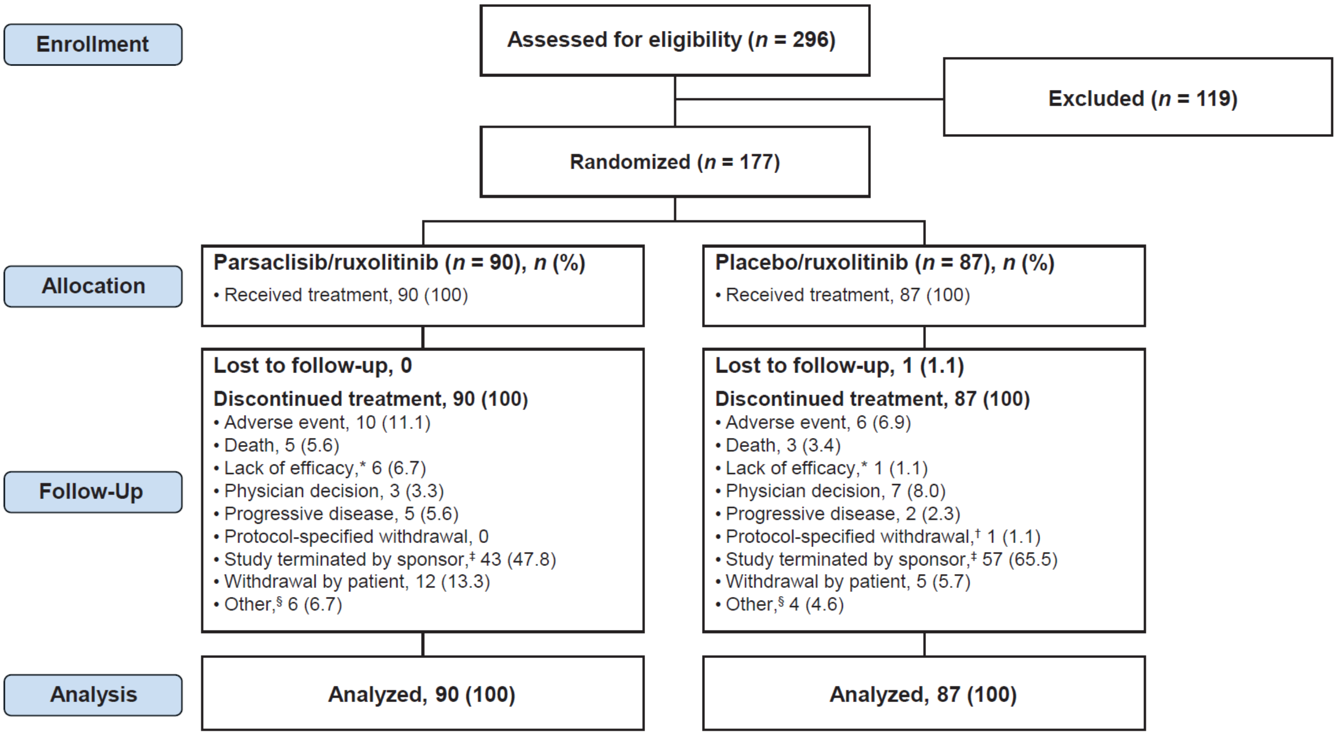

*Treatment termination due to lack of efficacy for the 1 participant in the placebo/ruxolitinib group occurred during the crossover period after the participant transitioned to parsaclisib/ruxolitinib. This participant later withdrew consent to continue receiving treatment.

†Participant did not meet inclusion criterion of palpable spleen of ≥5 cm and TSS of ≥10 at screening and was withdrawn from the study.

‡Treatment discontinuation reasons for 4 participants in the parsaclisib/ruxolitinib group and 1 participant in the placebo/ruxolitinib group who transitioned to a rollover study to receive continued treatment with parsaclisib/ruxolitinib were recorded as study terminated by the sponsor.

§Participants in the "other" category included those who transitioned to a rollover study to receive continued treatment with parsaclisib/ruxolitinib (5 participants in the parsaclisib/ruxolitinib group and 4 participants in the placebo/ruxolitinib group who had crossed over to parsaclisib/ruxolitinib).

TSS, total symptom score.

Supplemental Table 1. Summary of ruxolitinib exposure during the study

Variable	Parsaclisib/ruxolitinib		Placebo/ruxolitinib	
	Platelet count			
	50 to <100 × 10^9/L (n = 31)	≥100 × 10^9/L (n = 59)	50 to <100 × 10^9/L (n = 31)	≥100 × 10^9/L (n = 56)
Duration of treatment (days)				
Mean (SD)	298.6 (181.50)	329.7 (224.16)	293.7 (185.00)	324.9 (202.89)
Median	250.0	278.0	257.0	303.0
Min, max	22, 650	14, 898	14, 765	7, 874
Average daily dose (mg)				
Mean (SD)	20.9 (11.12)	27.8 (11.38)	22.5 (10.42)	26.8 (12.64)
Median	19.9	29.9	20.0	28.6
Min, max	6, 50	5, 50	9, 48	10, 50
Dose modifications n (%)				
Dose reduction	7 (22.6)	7 (11.9)	7 (22.6)	4 (7.1)
Dose interruption	5 (16.1)	2 (3.4)	3 (9.7)	4 (7.1)
Dose increase*	2 (6.5)	2 (3.4)	3 (9.7)	4 (7.1)

*Patients with a dose increase included those with a previous dose reduction due to AEs whose dose was reinstated to the original starting dose after resolution of any AEs.
AE, adverse event; SD, standard deviation.

Supplemental Table 2. Serious AEs occurring in at least two patients in any treatment group

**Event, n (%)**	**Parsaclisib/ ruxolitinib (n = 90)**	**Placebo/ ruxolitinib (n = 87)**	**Crossover from placebo to parsaclisib/ruxolitinib (n = 41)**
Pneumonia	8 (8.9)	2 (2.3)	2 (4.9)
COVID-19 pneumonia	5 (5.6)	1 (1.1)	0 (0.0)
COVID-19	3 (3.3)	0 (0.0)	1 (2.4)
Anemia	2 (2.2)	2 (2.3)	0 (0.0)
Platelet count decreased	2 (2.2)	0 (0.0)	1 (2.4)
Pneumonia influenzal	2 (2.2)	0 (0.0)	0 (0.0)
Pyrexia	2 (2.2)	0 (0.0)	0 (0.0)

AE, adverse event.

Supplemental Table 3. AEs of special interest

**Event, n (%)**	**Parsaclisib/ ruxolitinib (n = 90)**	**Placebo/ ruxolitinib (n = 87)**	**Crossover from placebo to parsaclisib/ruxolitinib (n = 41)**
CMV infection	10 (11.1)	0 (0.0)	2 (4.9)
Herpes zoster	4 (4.4)	4 (4.6)	2 (4.9)
Pneumonitis	2 (2.2)	0 (0.0)	1 (2.4)
ALT increased ≥5 × ULN	1 (1.1)	1 (1.1)	0 (0.0)
Diarrhea	1 (1.1)	2 (2.3)	1 (2.4)
Oral herpes	1 (1.1)	0 (0.0)	1 (2.4)
Genital herpes	0 (0.0)	0 (0.0)	1 (2.4)

AE, adverse event; ALT, alanine aminotransferase; CMV, cytomegalovirus; ULN, upper limit of normal.
